# Metabolic Flexibility and Mechanical Efficiency in Women Over-60

**DOI:** 10.3389/fphys.2022.869534

**Published:** 2022-04-06

**Authors:** Cristina Blasco-Lafarga, Jordi Monferrer-Marín, Ainoa Roldán, Pablo Monteagudo, Ivan Chulvi-Medrano

**Affiliations:** ^1^ Sport Performance and Physical Fitness Research Group (UIRFIDE), Physical Education and Sport Department, University of Valencia, Valencia, Spain; ^2^ Department of Education and Specific Didactics, Jaume I University, Castellon, Spain

**Keywords:** aging, cardiovascular health, exercise economy, gross efficiency, muscle power, physical exercise

## Abstract

**Purpose:** Aging deteriorates metabolic flexibility (MF). Moreover, recent studies show that glycolysis is barely increased despite impoverished lipid metabolism, in addition to increased relevance of muscle power in older adults. This study aims to analyze MF, i.e., fat and carbohydrates oxidation rates (FATox and CHOox), and the point of maximal fat oxidation (MFO), in a group of active women over-60. It also aims to delve into the role of power production and mechanical efficiency regarding MF. This will help to decipher their metabolic behavior in response to increasing intensity.

**Methods:** Twenty-nine women (66.13 ± 5.62 years) performed a submaximal graded cycling test, increasing 10 W each 3-min15-s, from 30 W to the second ventilatory threshold (VT_2_). Muscle power was adjusted with a Saris-H3 roller, together with a continuous gas analysis by indirect calorimetry (Cosmed K4b2). Pre and post-test blood lactate (BLa) samples were included. Frayn’s equations, MFO and CHOox_peak_ (mg/min/kg FFM) were considered for MF analysis (accounting for average VO_2_ and VCO_2_ in each last 60-s), whilst delta and gross efficiencies (DE%, GE%), and exercise economy (EC), were added for Mechanical Efficiency. Mean comparisons regarding intensities 60, 80 and 100% at VT_2_, completed the study together with correlation analysis among the main variables.

**Results:** MFO and CHOox_peak_ were small (6.35 ± 3.59 and 72.79 ± 34.76 g/min/kgFFM respectively) for a reduced muscle power (78.21 ± 15.84 W). Notwithstanding, GE% and EC increased significantly (*p* < 0.01) with exercise intensity. Importantly, coefficients of variation were very large confirming heterogeneity. Whilst muscle power outcomes correlated significantly (*p* < 0.01) with MFO (*r* = 0.66) and age (*r* = −0.62), these latter failed to be associated. Only GE% correlated to CHOox_peak_ (*r* = −0.61, *p* < 0.01) regarding mechanical efficiency.

**Conclusions:** Despite being active, women over-60 confirmed impaired substrates switching in response to exercise, from both FAT and CHO pathways. This limits their power production affecting exercise capacity. Our data suggest that decreased power with age has a key role above age *per se* in this metabolic inflexibility. *Vice versa*, increasing power seems to protect from mitochondrial dysfunction with aging. New studies will confirm if this higher efficiency when coming close to VT_2_, where GE is the more informative variable, might be a protective compensatory mechanism.

## Introduction

Metabolic flexibility describes the ability of an organism to respond or adapt to changes in metabolic or energy demands (Goodpaster and Sparks, 2017). Initially related to the reduced, or even supressed, free faty acids (FFA) muscle consumption after postabsorptive and postprandial conditions (Kelley et al., 1999), metabolic flexibility is currently considered a symptom of adaptability and mitochondrial health. In fact, it has become of paramount importance in the field of exercise metabolism, since large and many times abrupt changes in physiological conditions require metabolic flexibility to meet the variability in the energy demands (San-Millán and Brooks, 2017).

In terms of enhanced metabolic flexibility, exercise training promotes epigenetic (Rasmussen et al., 2014), transcriptomic (Raue et al., 2012), and proteomic (Hoffman et al., 2015) crosstalk’s in the skeletal muscle, constituting improvements in gene expression, fiber composition, skeletal signalling, as well as improvements in mitochondrial function, due to the increased AMPK activity (Jørgensen et al., 2006) or Sirtuin-3 activation (Jing et al., 2011). At the opposite end, this ability to provide and regulate substrates deteriorates under different metabolic conditions such as aging, where there are impoverishments as the gradual decrease in the fat free mass (FFM), the decline in the cardiorespiratory fitness and the impaired fat oxidation capacity during exercise (Toth and Tchernof, 2000). This deterioration may be a consequence of the impaired muscle mass with aging, or even the aging process *per se*, but it is also linked to the physical activity reduction along the lifespan, what aggravates mitochondrial dysfunction, an aging hallmark (López-Otín et al., 2013).

Mitochondrial perturbations downstream to changes in cellular dynamics associated with loss of muscle quality and increased oxidative stress (Peterson et al., 2012). Mitochondrial dysfunction is thus linked to muscle ailments and increased fatigue in older adults, as shown by Santanasto et al. (2015). Importantly, these conditions causing impaired metabolic flexibility yield to a mitochondrial evolution towards a more glycolytic phenotype (Hyatt and Powers, 2021). Nevertheless, this evolution shows a smaller increase in the older adults despite their impoverished lipid metabolism (Braganza et al., 2019; Monferrer-Marín et al., 2022), what becomes a limiting factor for physical exercise.

In this context, Monferrer-Marín et al. (2022) have introduced muscle power as a control variable in their recent pilot study about the association of metabolic flexibility and aging in older women, downplaying the link between age and metabolic inflexibility. According to these authors, both carbohydrates and fat oxidation rates (CHOox and FATox respectively) confirm to be lower in this population. However, the main role of muscle power when talking about fat oxidation suggests a shielding effect of exercise over the metabolic dysfunction in these women. The deterioration of the point of Maximal Fat Oxidation (MFO) that accompanies aging could be due to the low muscle power outputs at higher intensities, almost blunting the old women capacity to pedal at FATmax intensities. Moreover, these impoverished metabolic conditions correlate in turn with low levels of exercise efficiency in older adults, an additional limiting factor which may hinder normal activities of daily living (Broskey et al., 2015). Up to our knowledge, the role of efficiency and muscle power regarding metabolic flexibility in older women remains unknown and opens new questions about their cardiovascular health and the benefits of exercise in the older populations.

Of uttermost importance, exercise efficiency is a complex term determined by the coordinated addition of the cardiorespiratory, metabolic, neuromuscular, and biomechanical efficiencies (Barnes and Kilding, 2015). Cardiorespiratory and metabolic efficiencies are related to the supply of oxygen to force-producing muscles and the resynthesis of adenosine triphosphate in them, whereas neuromuscular and biomechanical efficiencies reflect the interactions between the neural and musculoskeletal systems (Carlsson et al., 2020). This complexity explains that efficiency assessment encompasses several indexes which vary with the different protocols -e.g., Delta Efficiency (DE), Gross Efficiency (GE) and exercise economy (EC)-, as well as some inconsistencies regarding exercise in older adults.

Whilst Gaesser et al. (2018) reported that older subjects had as well lower, as equal, or even higher cycling efficiency compared to younger subjects, more recently Grevendonk et al. (2021) has shown impaired efficiency in older adults with equal physical activity, what highlights the influence of age. Broskey et al. (2015) also found this efficiency deterioration with aging, including an efficiency correlation with mitochondrial content and function. However, Rosenbaum et al. (2003) found increases in efficiency parallel to increases in oxidative phosphorylation capacity with exercise. And nonetheless the complexity and disparity in the assessment of efficiency, it seems that continued exercise combats disease and mitochondrial disfunction by increasing mitochondrial content and mitochondrial function/efficiency despite advanced age, as reflected by the higher efficiency values observed in older athletes (Amati et al., 2011). Also Villelabeitia-Jaureguizar et al. (2019) found efficiency improvements after high intensity interval training in older adults suffering from severe cardiovascular pathologies. Exercise seems to matter, but to highlight, none of these scarce studies included only older women, despite (Maunder et al., 2018) showed gender differences such as that women had higher fat oxidation when normalizing to FFM.

Therefore, physical capacity (i.e., muscle power) and efficiency are important in the maintenance of cardiovascular/metabolic health in older individuals, what might influence their metabolic flexibility. In addition, there is scarcity of studies exclusively with women, despite their differences in strength and power production compared to men might be hiding some information regarding their metabolic flexibility and its association with exercise. This research aims to go further in the analysis of the metabolic flexibility/inflexibility in a group of active women over-60 years, as well as to delve into the meaning of different efficiency indicators such as GE, DE and EC in this population, largely forgotten by the physiology of exercise. Of equal importance, the study aims to better understand the influence of age, but also muscle power and efficiency in this indirect assessment of mitochondrial health.

As a first hypothesis, women over-60 years of age will present alterations on metabolic flexibility variables, which will be conditioned by muscle power, besides the influence of age. As a second hypothesis, regarding the efficiency variables, it is expected that they may show an association with both, age and metabolic flexibility indicators, correlations which will be also sensible to muscle power.

## Material and Methods

### Participants

**FIGURE 1:**
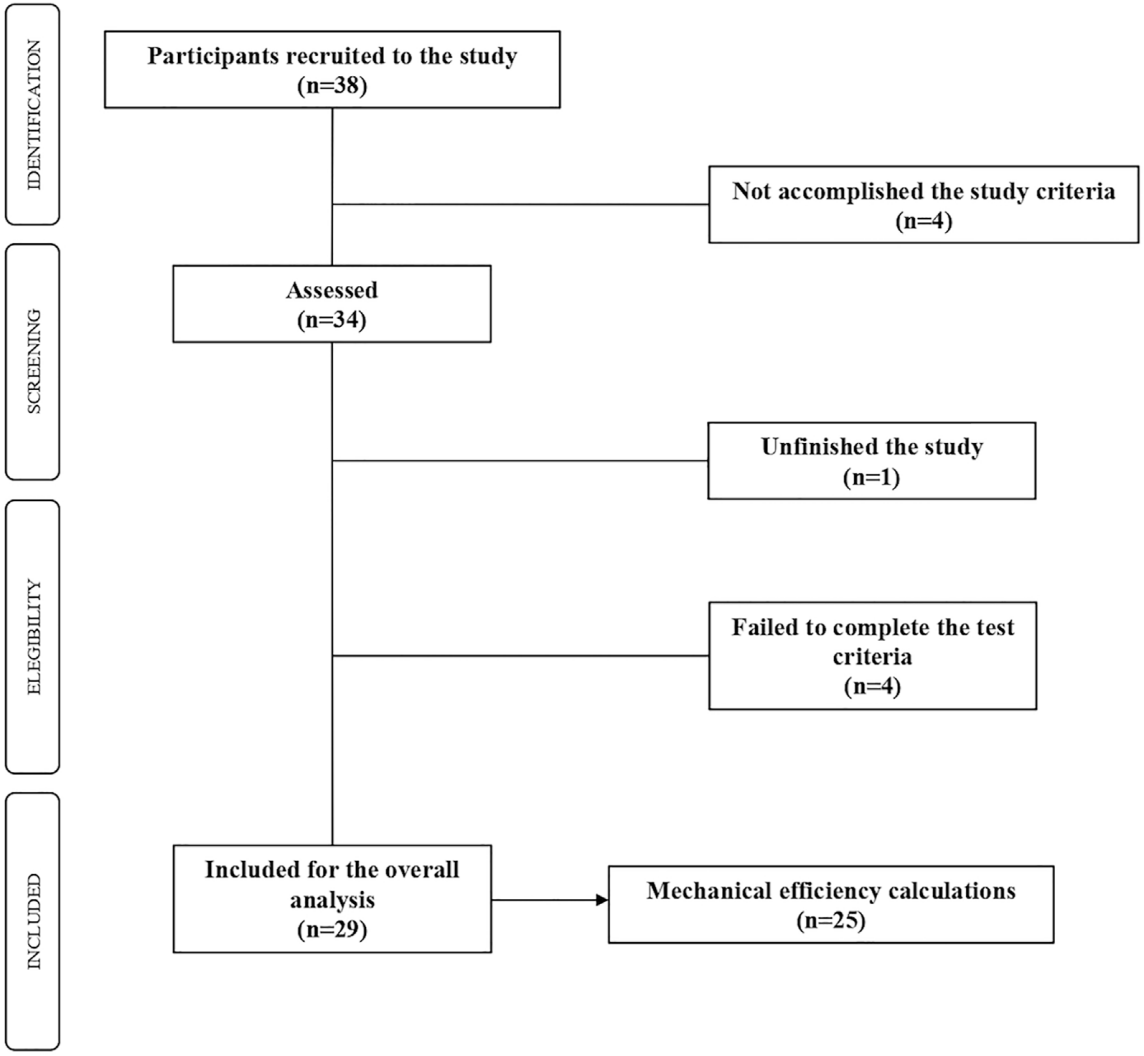
Flowchart of the participants.

Following similar studies in the topic (Amaro-Gahete et al., 2018; Maunder et al., 2018), the sample size was set over the 30 participants and a 10% drop out. Thirty-eight active women volunteered to participate in the study. Inclusion criteria were to be female over-60 years, moderately active according to the International Physical Activity Questionnaire (IPAQ), and absence of any medical contraindication for physical exercise according to the physical activity readiness questionnaire (PAR-Q). Exclusion criteria were to be diagnosed insulin resistance, the consumption of drugs (e.g., beta-blockers) that limit or condition the practice of physical exercise, and the non-compliance with any of the inclusion criteria. As displayed in the flow chart (Figure 1), 29 of these women completed the graded test and comprised the final sample. Since four of this 29 women did not reach the minimum number of intensity-increases to represent their evolution with respect to efficiency (GE%, DE%), the mechanical analysis were reduced to 25 women.

All women were fully informed about the protocol and its possible risks and benefits, signing the written consent. The protocol was conducted according to the Declaration of Helsinki and was approved by the Science ethical committee of the local University (H105715353921).

### Experimental Design

**FIGURE 2:**
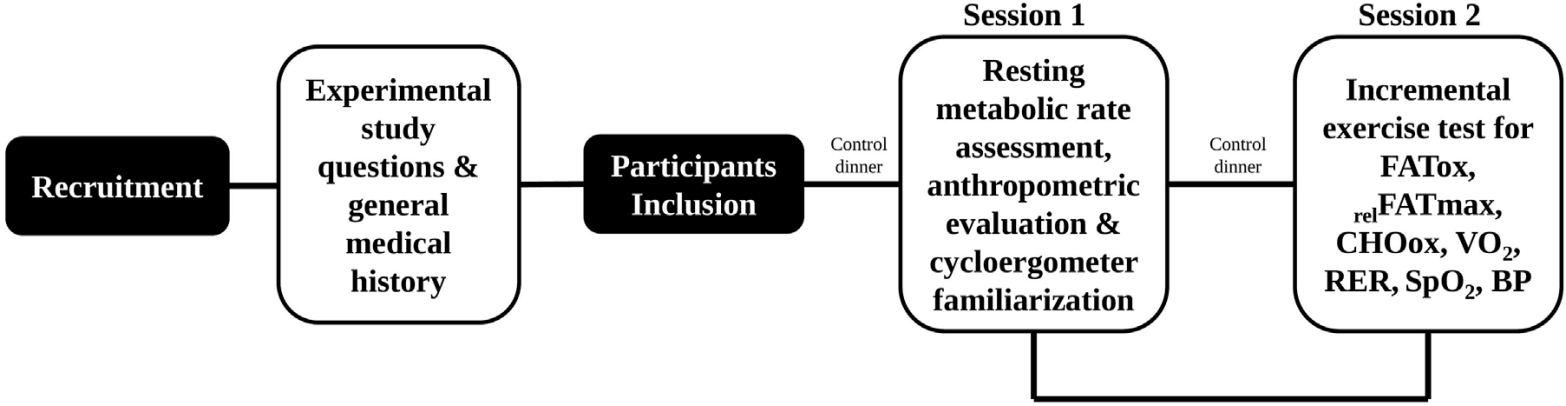
Stepwise experimental design. FATox, fat oxidation; _rel_FATmax, the intensity at which MFO point is reached; CHOox, carbohydrate oxidation; VO_2_, oxygen consumption; RER, respiratory exchange ratio; SpO_2_, oxygen saturation; BP; blood pressure.

The study, conducted between March and December 2021, followed a single centre, cross-sectional design. Once after the first telephone interview and recruitment, the protocol included two individual measurement sessions (Figure 2).

Women were told to refrain from strenuous exercise 24 h before the test and to follow their usual diet, maintaining its macronutrient composition and energy content except for the pre-test dinner, where they should ensure carbohydrates enough to avoid overly condition the oxidation rates. They were also asked to abstain from caffeine 1.5 h and to fast at least 2 h before the test. These guidelines, which helped to homogenize the diet and the women CHO availability (San-Millán and Brooks, 2017), were repeated on both days in the study. In addition, women were instructed to arrive at the laboratory well rested and were asked to travel by car or public transport.

Body composition was assessed by means of the stadiometer SECA 222 (Hamburg, Germany) for the height, and the bioimpedance bascule Tanita DC-430 MA S scale (Tokyo, Japan) for weight and FFM determination. These outcomes (body weight and FFM) were further used to normalize some parameters such as power or substrate oxidation.

### Exercise Test

In order to determine the oxidation rates, what requires long-lasting stages (Amaro-Gahete et al., 2019), intensity was increased 10 W (W) every 3-min 15-s by means of the Roller Saris H3 (CycleOps Hammer Direct Drive Trainer, Saris, Madison, United States) and the Rouvy application (VirtualTraining, Vimperk, Czech Republic). The test started at 30 W to allow women complete a minimum overall duration, and the 15-s were added in every stage due to the mechanical limitations of this population (Yamauchi et al., 2010). The increase in intensity was continuously monitored and adjusted with the help of an iPad tablet (Apple, Cupertino, CA, United States) -*see* Monferrer-Marín et al. (2022) for more details-.

Notably, heart rate (HR), adapted rating of perceived exertion (RPE) Borg scale 1–10 (DaSilva-Grigoletto et al., 2013) and Visual Analogue pain Scale (VAS) were controlled every 1-min 30-s along the test. The protocol aimed to reach the second ventilatory threshold (VT_2_), determined by at least two of the three following criteria: RER >1.1 (Diefenthaeler et al., 2017), peak of HR (HR_peak_) >80% HR_max_ (Karapetian et al., 2008), and/or RPE >6 (Seiler and Kjerland, 2006; Diefenthaeler et al., 2017). Whenever a VAS>5 and/or oxygen saturation (SpO_2_%) <92%, the women were invited to end the test to ensure a comfortable and secure testing session. Then, after the cessation of the incremental protocol, women were told to pedal again at 30 W for recovery, and blood lactate (BLa) was extracted at minutes 3 and 5 for Bla_peak_, while still pedalling (Lactate Scout, SensLab GmbH, Leipzig, Germany).

VO_2_ and VCO_2_ were measured by indirect calorimetry using the K4b2 metabolic unit (Cosmed, Rome, Italy). The online gas analysers were carefully calibrated with an automated volume calibration and with a gas mixture recommended by the manufacturer prior to the start of each test. As above mentioned, SpO_2_ was monitored along the whole protocol to guarantee the security of the participants (Wristox2 3100 pulse-oximeter: Nonin Medical, Plymouth, MN, United States), together with HR, which was recorded continuously with a Polar H10 band (Polar Electro Oy, Kempele, Finland). The assessments also included a pre-test glycaemia control, which was registered by means of a flash glucose monitoring system (FreeStyle Libre, Abbott Diabetes Care, Witney, United Kingdom); and a pre and post-test blood pressure registers (BP; Omron M6 sphygmomanometer (HEM-7420, Omron Healthcare, Kyoto, Japan).

### Calculations

#### Metabolic Flexibility and Whole-Body Fat Oxidation Kinetics

Frayn’s equations were used for the calculation of the whole-body oxidation rates, with the assumption of urinary nitrogen excretion rate as negligible (Frayn, 1983), at the intensities 60, 80 and 100% of the individual peak of power in the test (P_60_, P_80_ and P_100_ respectively) (Monferrer-Marín et al., 2022).

$$\mathrm{FATox}\left(g/\min\right)=1.67{\mathrm{VO}}_{2}\left(L/\min\right)–1.67{\mathrm{VCO}}_{2}\left(L/\min\right)$$

$$\mathrm{CHOox}\left(g/\min\right)=4.55{\mathrm{VCO}}_{2}\left(L/\min\right)–3.21{\mathrm{VO}}_{2}\left(L/\min\right)$$

The average of the last 60-s in each intensity was considered (Amaro-Gahete et al., 2019). Thereafter, MFO and the relative FATmax (_rel_FATmax; exercise intensity that elicits the MFO, expressed as a percentage of the VO_2_peak) were determined considering both, the total body weight (g/min) as well as the FFM value (g/min FFM). This latter may be more appropriated when making comparisons by sex (Amaro-Gahete et al., 2018; Amaro-Gahete et al., 2021; Frandsen et al., 2021).

### Mechanical Efficiency and Economy

To have a good overview on efficiency, gross efficiency and delta efficiency were calculated, followed by the analysis of cycling economy. Noteworthy, these measurements benefit from long lasting submaximal test where intensities are fixed for a minimum duration, looking for VO_2_ stability in the exercise intensity under assessment (De Koning et al., 2012). However, GE has confirmed to be robust in shorter graded exercise stages whenever they look for steady state exercise and RER is ≤1.0 (De Koning et al., 2012); and DE has also been extracted from shorter graded test (Santalla et al., 2009).

For the calculation of GE and DE, mechanical power output and metabolic power input (MPI) were prior determined and expressed both in kcal·min, using again the mean of VO_2_ and RER values over the last 60-s of each stage in the graded test (Santalla et al., 2009). Following these authors, those stages with RER>1.0 were excluded.

$$\mathrm{Mechanical}\;\mathrm{power}\;\mathrm{output}\;\mathrm{in}\;\mathrm{Kcal}\cdot \min=\frac{\mathrm{Power}\left(W\right)}{69.7\left(W\cdot \mathrm{kcal}-1\cdot \min-1\right)}$$

$$\mathrm{Metabolic}\;\mathrm{power}\;\mathrm{input}\;\mathrm{in}\;\mathrm{Kcal}\cdot \min={\mathrm{VO}}_{2}\left(L/\min\right)\cdot \left(1.2341\cdot \mathrm{RER}+3.8124\right)\;$$

GE was then calculated as the ratio between the external mechanical work developed at a certain intensity and its metabolic cost -in %-. That is, the percentage of the metabolic production of energy used to produce the work at that intensity during exercise (GE%).

$$\mathrm{GE}\left(\%\right)=\left(\frac{\mathrm{Mechanical}\;\mathrm{power}\;\mathrm{output}}{\mathrm{Metabolic}\;\mathrm{power}\;\mathrm{input}}\right)\cdot 100$$

Once the participants remained at least two stages below a RER = 1, their GE% values were graphically plotted.

DE, expressed in percentage (%), was then extracted from the linear regression between the energy expenditure required at each load (*X* axis, in kcal·min^−1^) and the external mechanical power output (*Y* axis, in kcal·min^−1^) (Santalla et al., 2009).

Later on, economy (EC) was additionally calculated according to the Moseley and Jeukendrup (2001) equation, where the economy (KJ·L^−1^) equals the ratio between the mean power output in the last 60 s and the mean steady-state oxygen uptake (L·min^−1^) of each step of the test.

$$\mathrm{Economy}\left(\mathrm{KJ}\cdot L^{-1}\right)=\frac{\mathrm{Power}\left(W\right)}{{\mathrm{VO}}_{2}\left(L\cdot \min^{-1}\right)}$$

### Statistical Analysis

Data analyses were performed with the SPSS statistics package version 25 (IBM SPSS Statistics for Windows, Chicago, IL, United States). As a first descriptive approach, mean, standard deviation (SD) and coefficient of variation (CV) were calculated to describe the main variables (metabolic flexibility, performance, mechanical efficiency, and economy indicators). After testing for normality (Shapiro–Wilks), the Friedman’s ANOVA test, followed by Wilcoxon matched pairs *post-hoc* tests were applied for GE% and EC outcomes corresponding to the intensities 60, 80 and 100% with regard to the individual peak of power in the test (P_60_, P_80_ and P_100_ respectively). Data from these intensities have been represented in violin plots using the GraphPad Prism^®^ 5 (Version 5.01, GraphPad Software, Inc., La Jolla, CA, United States). In addition, the slope of the line or DE%, showing the relationship between mechanical efficiency and MPI, was plotted. Later on, Spearman’s or bivariate correlations were performed among all these key markers. As normality was assumed only for MFO, _rel_FATmax and CHOox, Pearson bivariate correlations were performed in these cases. Finally, the effect size for paired mean differences was calculated by means of the Cohen’s d, where the effect was considered small (*d* = 0.2–0.4), medium (*d* = 0.5–0.7) or large (*d* = 0.8–2.0) according to Cohen (1988). The significance level was set at <0.05.

## Results

Table 1 displays the main body composition and physiological characteristics of the sample (upper section), and the cardiovascular and performance indicators in the test (lower section). MFO was small (6.35 ± 3.59 g/min/kgFFM), similarly to VO_2_peak (17.32 ± 7.94 ml/kg/min), although this was a submaximal outcome closed to the second ventilatory threshold. This latter resulted in a high relative to exercise cessation _rel_FATmax (70.73 ± 22.02 %VO_2_peak).

**TABLE 1:** Physical characteristics of the participants and Cardiovascular responses in the test.

	Mean ± SD	CV (%)
Anthropometric and physiological variables		
Age (years)	66.13 ± 5.62	8.50
Weight (kg)	65.16 ± 11.07	16.99
BMI (kg/m^2^)	25.89 ± 2.85	11.01
FFM (kg)	40.99 ± 5.71	13.89
Body fat (%)	37.64 ± 5.93	15.57
SBP (mmHg)	126.42 ± 15.66	12.39
DBP (mmHg)	78.45 ± 9.56	12.19
HR_baseline_ (bpm)	67.58 ± 8.96	13.26
Cardiovascular and performance parameters		
HR_pre_ (bpm)	69.17 ± 9.15	13.23
HR_peak_ (bpm)	136.33 ± 19.69	14.44
%HR_peak_ (bpm)	84.08 ± 11.09	13.19
VO_2peak_ (ml/kg/min)	17.32 ± 7.94	45.84
_rel_FATmax (%VO_2peak_)	70.73 ± 22.02	31.13
MFO (g·min·kgFFM)	6.35 ± 3.59	56.54
CHOox_peak_ (g·min·kgFFM)	72.79 ± 34.76	47.75
BLa_pre_ (mmol·L)	1.62 ± 0.92	56.79
BLa_peak_ (mmol·L)	7.06 ± 3.29	46.60
Power_peak_	78.21 ± 15.84	20.25

Data are presented as the mean ± SD and CV (%). BMI, body mass index; FFM, fat-free mass; SBP, systolic blood pressure; DBP, diastolic blood pressure; SpO_2_, oxygen saturation; HR_pre_, heart rate pre-test; HR, heart rate; %HR_peak_, percentage of its HR_peak_; VO_2peak_, oxygen consumption peak; _rel_FATmax, the intensity at which MFO point is reached regarding to VO_2peak_; MFO, maximal fat oxidation relative to FFM; CHOox_peak_, maximal rate of carbohydrates oxidation in the test relative to FFM; BLa, Blood lactate.

**FIGURE 3:**
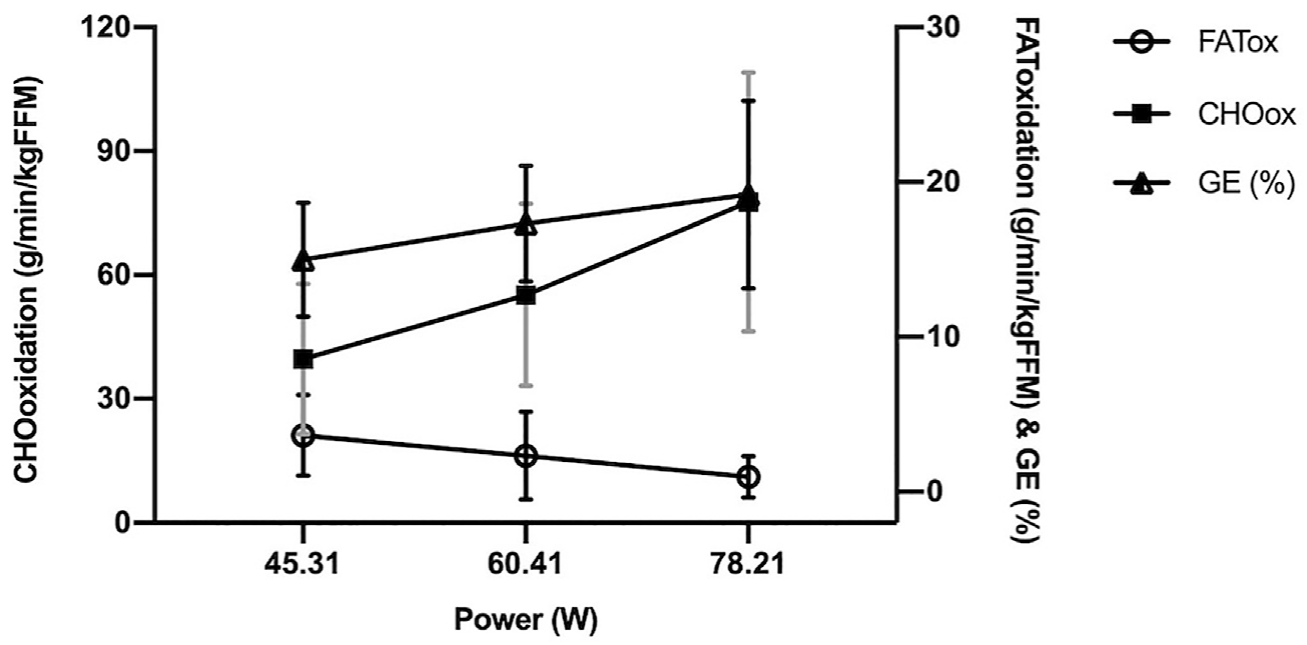
Oxidation rates and gross efficiency evolution as a function of graded exercise (power). FATox, fat oxidation; CHOox, carbohydrate oxidation; GE, gross efficiency. The x axis shows mean values for P_60_, P_80_ and P_100_, 60, 80 and 100% respectively from the peak of muscle power in the test.

As shown by Figure 3, the substrates evolution along the test reflected a limited capacity to use both, FATox and CHOox at the intensities P_60_, P_80_ and P_100_, with the consequence of low muscle power outputs despite being active women (mean power: 45.31 ± 12.79 W, 60.41 ± 17.05 W and 78.21 ± 15.88 W respectively). In addition, Figure 3 revealed a similar trend between GE% and CHOox, shown in reverse regarding FATox.

**FIGURE 4:**
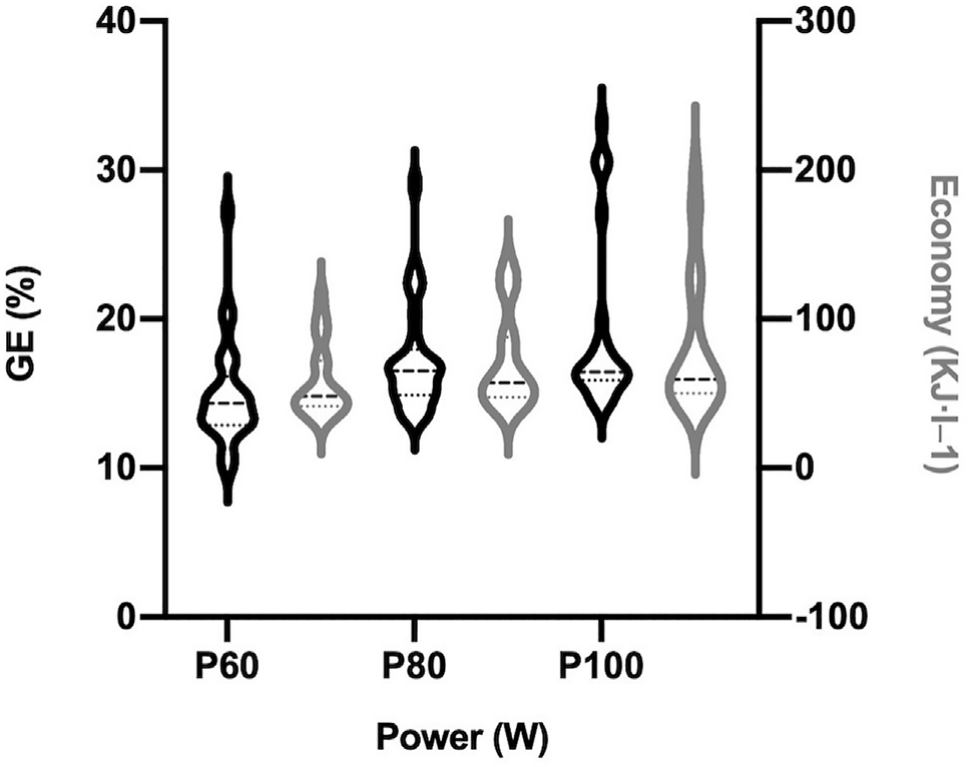
Mechanical efficiency markers in three key points of test. Gross Efficiency (%) and exercise Economy (KJ·L^−1^) distribution in violin plots along the incremental test. P_60_ (power at 60% of Power_peak_), P_80_ (power at 80% of Power_peak_), and P_100_ (Power_peak_) representing the three key points calculated from the peak of muscle power in the test.

GE% showed larger average value than expected in P_80_ and P_100_, as well as a great heterogeneity (Figure 4). Friedman tests revealed significant differences regarding intensity (P_60_, P_80_ and P_100_; χ^2^ = 26.88, N = 25; *p* < 0.01), and Wilcoxon *post hoc* tests confirmed these differences between them all (Table 2): P_60_ vs P_80_ (*p* < 0.01) and P_100_ (*p* < 0.01); P_80_ vs P_100_ (*p* = 0.03). Therefore, efficiency measured as GE% increased significantly along the test, with the better outcomes at its end, closed to the second ventilatory threshold of the participants.

**TABLE 2:** Gross efficiency and exercise economy along a submaximal cycling graded test.

		P_60_	P_80_	P_100_
Mean ± SD	GE	14.99 ± 3.67^a^	17.33 ± 3.73^b^	19.19 ± 6.05^b^
	EC	59.35 ± 24.57^a^	71.79 ± 32.33^a^	83.33 ± 47.31^a^

Data are presented as the mean ± SD. GE, gross efficiency; EC, exercise economy; P_60_: 60%, P_80_: 80%, and P_100,_ 100% of the peak of power in the test.

a *p* < 0.01 between groups (Wilcoxon *post-hoc* test).

b *p* < 0.05.

Figure 4 also showed this great heterogeneity in EC. Friedman tests revealed again significant differences regarding intensity (P_60_, P_80_ and P_100_; χ^2^ = 44.90, *N* = 25; *p* < 0.01), and Wilcoxon *post hoc* tests confirmed all these differences (*p* < 0.01, Table 2).

Therefore, both efficiency and economy increased as intensity augmented (Figure 4).

**FIGURE 5:**
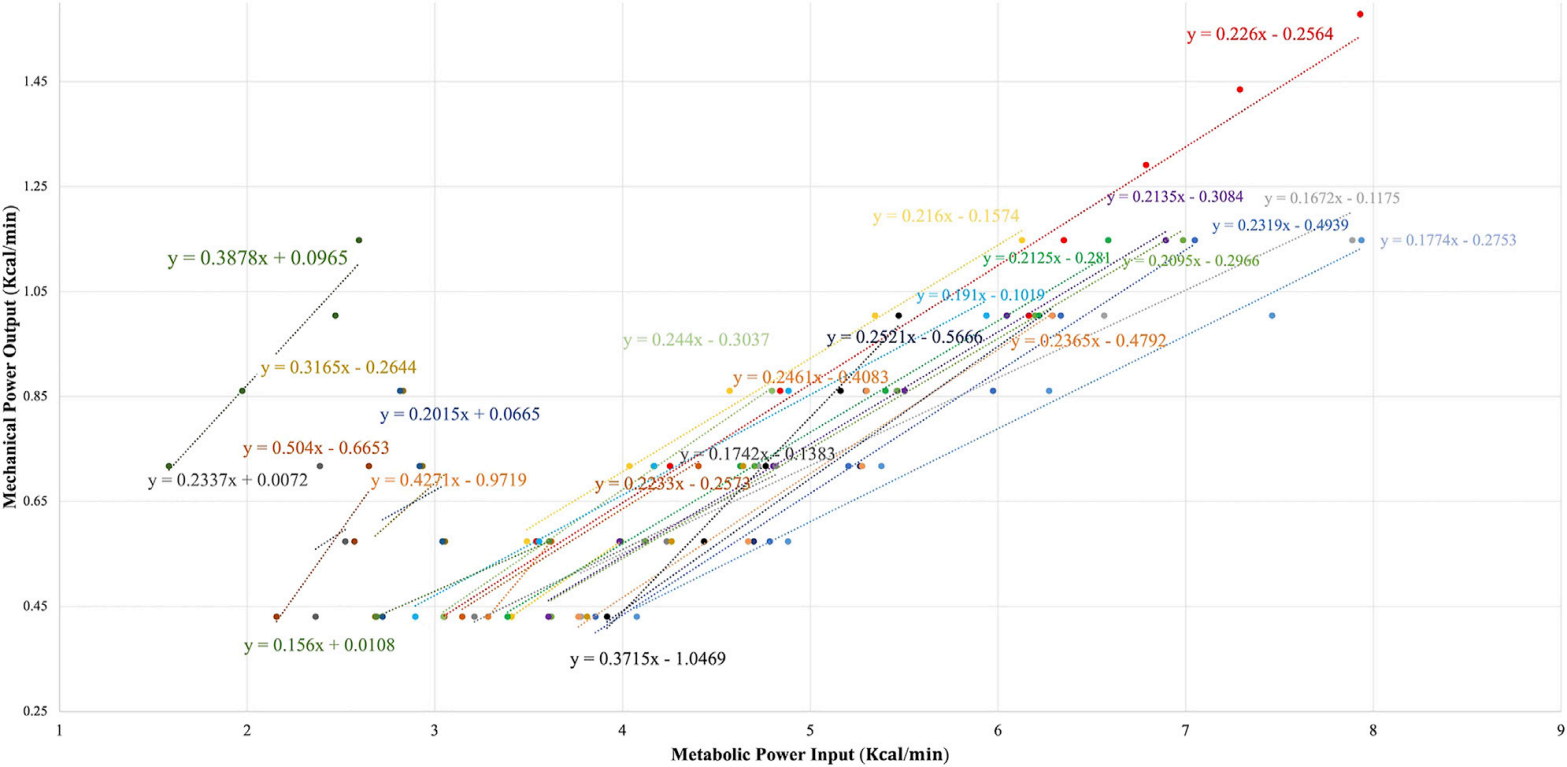
Delta efficiency of all the participants including the RER ≤1 stages (*N* = 25).

As reflected by Figure 5, the big individual differences persisted in the DE% slope of all those participants who allowed its calculation (mean DE% = 24.85 ± 8.69%). Moreover, there was a bit larger heterogeneity in DE% compared to GE% at P_100_ (GE%_CV_: 31.53% vs. DE%_CV_: 34.97%).

With regard to the correlation analysis, as displayed in Table 3, there was a non-existent relationship between age vs. MFO (*r* = −0.36: *p* = 0.12), and age vs. CHOox_peak_ (g/min/kgFFM) (*r* = −0.15: *p* = 0.44). In addition, it appears to be a significant and large dependence of MFO, which is reduced to just a trend on CHOox_peak_, regarding the peak of muscle power in the test: MFO vs Power_peak_ (*r* = 0.66; *p* < 0.01); CHOox_peak_ vs Power_peak_ (*r* = 0.35; *p* = 0.06). In turn, power also showed a large and significant negative association with age (*r* = −0.62; *p* < 0.01).

**TABLE 3:** Correlation’s coefficients among the key markers in the study of the metabolic flexibility.

Spearman coefficient	MFO	Age	Power_peak_
MFO	—	−0.36	0.66^a^
Age	−0.36	—	−0.62^a^
Power_peak_	0.66^a^	0.62^a^	—

MFO, maximal fat oxidation; Power_peak_, 100% of the peak power in the test, closed to the second ventilatory threshold.

a *p* < 0.015.

MFO, in order, displayed a significant, positive and strong association with MPI (*r* = 0.74; *p* < 0.01), which was medium with respect to BLa_peak_ (*r* = 0.40; *p* < 0.05). Age, for its part, was also associated significantly, medium and negatively with this BLa_peak_ (*r* = −0.54; *p* < 0.01), just as with the level of physical activity (IPAQ) (*r* = −0.54; *p* < 0.01).

Regarding muscle power, Power_peak_ was associated with a multitude of parameters of relevance in this study, being this association large and positive with MPI (*r* = 0.67; *p* < 0.01) and BLa_peak_ (*r* = 0.66; *p* < 0.01), in addition to the above-mentioned associations with MFO (*r* = 0.66; *p* < 0.01) and age (*r* = -0.62; *p* < 0.01). This associations got reduced but was still medium with respect to the level of physical activity (IPAQ; *r* = −0.51, *p* < 0.05), _rel_FATmax (*r* = −0.58; *p* < 0.01), being negative in the latter. Notably, despite they were still significant, there was a drop in the correlation coefficients when normalizing power, both with the kg of total body mass and fat-free mass (being lower the reduction with this latter), except in the association regarding MPI, IPAQ and _rel_FATmax.

Also noteworthy, none of these three variables (age, MFO and Power_peak_) showed any association with efficiency and economy (GE%, DE% and EC), with the only significant association for these latter for GE% vs CHOox_peak_ (*r* = −0.61, *p* < 0.01).

## Discussion

As a main finding, the study confirms the trend to metabolic inflexibility in older women, with small substrates availability and slow interchange between FATox and CHOox secondary to the increase of intensity in a graded test. Also as hypothesized, muscle power showed an important influence in this metabolic response to exercise, not only on MFO but also on lactate and most of the main variables in the test, even on CHOox_peak_ where the association was just a trend. Contrary to expectations, nor MFO nor CHOox_peak_ were associated with age, downplaying the link between age and metabolic inflexibility. These results confirm the paramount importance of the maintenance of muscle power in women over-60.

Moreover, despite this greater metabolic rigidity, active women over-60 managed to be increasingly more efficient throughout the test, even when coming close to VT_2_. This might help to compensate and protect from the inflexibility related to the loss of muscle power with age, with the gross efficiency as the most informative variable for this mechanical efficiency. To our knowledge, this is the first study to include power in the analysis of mechanical efficiency in relation to metabolic flexibility in older women.

Despite the deterioration process, it is not aging that determines the drop in MFO, but muscle power, which seems to indicate that those women who preserve power, reaching a higher performance in the test, preserve the ability to oxidize more fat at lower intensities. That is, a short motor performance in the test might punish the FFA oxidation by limiting the so-called low intensities. Noteworthy, larger muscle power does not guarantee a more efficient response to intensity up to VT_2_, what could be explained by the large number of factors that determine exercise efficiency, beyond metabolic or neuromuscular factors (Barnes and Kilding, 2015).

Also noteworthy, the impaired MFO observed in our older women has been previously shown in both sexes (Maunder et al., 2018). However, the consequences could be worse in the female population because they already display lower values at baseline (Amaro-Gahete et al., 2018), even closer to conditions with glycolytic dependence. MFO is actually related to a lower CD36 content or lower CS activity (Maunder et al., 2022), intimately linked to atherosclerotic processes (Tian et al., 2020) or tumor defense (Wang et al., 2020), as well as poorer physical performance (Maunder et al., 2022). Therefore, more studies focusing specifically on women should be promoted regarding aging and their cardiometabolic profile in exercise, both in healthy and pathology populations, since research and knowledge is still scarce (Amaro-Gahete et al., 2021).

On the other hand, there was no correlation between MFO and mechanical efficiency, or even between muscle power and mechanical efficiency, in contrast to what was expected. Notwithstanding, GE% correlated negatively with CHOox_peak_. Although lower CHOoxpeak reveals that the glycolytic pathway is also affected in elderly women, a very low capacity to use fat gives the CHOox pathway a paramount importance in our sample, which in addition displays that inverse association with GE%. Already Santalla et al. (2009) showed that the larger capacity to get VO_2_ and power production was inversely related to efficiency (DE%) in elite athletes, suggesting that these larger outputs were related to the better use of the Type II fibers, and not the more oxidative Type I. Similarly, our older active women seem to be able to use larger CHOox to get additional watts, inversely to the increase in efficiency. Being more efficient by reducing carbohydrates oxidation rates might have allowed the women to go on along the exercise protocol compensating carbohydrates paucity, thus becoming a protective compensatory mechanism.

To account, given that the aging process is associated to a drop in fibers type II, expressed as the percentage of each type of fiber regarding the total (Hester et al., 2022), the glycolytic pathway might have been punished, being sometimes unable to compensate the impoverished lipid metabolism (i.e. low MFO). A worse fiber type I/type II ratio is indeed related to poorer efficiency (Horowitz et al., 1994). Nevertheless, the more powerful and active old women in our study, as reflected by the association between muscle power and IPAQ, got also the greater CHOox_peak_, what could point to a larger preservation of type II fibers in these active women (Phu et al., 2015). Hence, muscle power seems to allow the older active women to go further in the test, with better metabolic flexibility responses, but this does not ensure their larger efficiency. Nonetheless, larger efficiency as measured by gross efficiency is effectively related to a reduced carbohydrates oxidation.

Also to account, this correlation between GE and CHOox_peak_ might have been affected by the anticipated RER≈1, consequence of the early predominance of carbohydrates, likely due to mitochondrial dysfunction (Zia et al., 2022), the long stages in the protocol (>3-min), and/or the limited strength in older women (Monferrer-Marín et al., 2022). Indeed, some factors concerning large protocols induce fatigue in less fit, inactive or older population as compared to shorter protocols (Chura et al., 2012). Moreover, this peculiarity in the RER lead to a MPI impoverishment and VO_2_peak, values pickled by the limited extension of the test, along with poor CHOox_peak_, what might have finally resulted in an unreal preserved efficiency. Likewise, DE is also affected by this phenomenon, because the early glycolytic RER supposes a shorter test which results in a steeper slope. In turn, this marker shows great heterogeneity, a fact that may condition the existing correlations, so DE% should be taken with caution (Blasco-Lafarga et al., 2021).

Since Broskey et al. (2015) found a moderate association of mitochondrial content with mechanical efficiency, new studies are needed to delve into the possibility and physiological meaning of these compensating mechanisms, including muscle biopsies and/or net efficiency calculations, as in Broskey et al. (2015). These future studies should necessarily consider muscle power and age. In addition, it is necessary to reconsider the FATmax outcomes, _rel_FATmax in our study, probably referring the impact of the above cited early glycolytic RER, which was one the criteria delimiting the end of the test (RER>1). VO_2_peak was likely far from the VO_2_max in the more fit women. Therefore, the intensity at which MFO is reached in percentage regarding this mean VO_2_peak in our active women (70.73% of VO_2_peak) was higher than expected, even higher than FATmax in physically active younger women; e.g. 50%VO_2_max in Maunder et al. (2018).

To conclude with the analysis of mechanical efficiency, GE% and DE% did not correlate between them at Power_peak_, as already noted in previous works (Broskey et al., 2015). Whilst DE informs about the averaged mechanical efficiency of an isolated musculoskeletal system, that is, the test as a whole, GE expresses the mechanical efficiency in any segment of this test, considering the metabolic behavior in the segment as a whole body (Matomäki et al., 2019). In addition to the above-mentioned limitation of DE in order to evaluate the short tests in these populations, this index displayed larger heterogeneity and has shown to be either sensible to the testing protocols (De Koning et al., 2012). Conversely, GE which reflected association with CHOox_peak_, allowed the comparison of efficiency responses to incremental exercise and shared a similar behavior to exercise economy, becoming the most appropriated in our study for the analysis of the mechanical efficiency. Indeed De Koning et al. (2012) already pointed that GE% was a robust marker of mechanical efficiency as long as the exercise intensity was performed in a steady state increasing intensity until RER ≤1.0. Of importance, the fit sample of young cyclists in De Koning et al. (2012) increased GE% up to VT_1_ and then remained stable, whilst women in our study, with little or blunted VT_1_ intensities, increased GE% until VT_2_. These authors also failed to associate muscle power and efficiency.

In summary, metabolic flexibility calculated by indirect calorimetry along an incremental test, is confirmed as an appropriated and secure method to study indirectly mitochondrial function, allowing to know the reduced capacity of older women to cope with increased energy needs, both in exercise and daily life (Romanello, 2020). Older women capabilities might be greatly impaired if FATox and CHOox were to fall sharply. In turn, our results give light to the key role of muscle power in the maintenance of metabolic flexibility, thus confirming the link between muscle power and mitochondrial health.

Muscle power has already shown to be highly relevant for life expectancy and quality of life in older adults (Zambom-Ferraresi et al., 2015), becoming thus a determining factor in the health of these older women. The present study confirms that muscle power in situations of metabolic demand is equally important, so its preservation confirms to be a main target against age-related declines whatever the physiological approach. Besides, despite the deterioration with age, muscle power is very sensible to training (Carneiro et al., 2021), so its improvement might easily help to enhance important health factors such as MFO, CHOox_peak_, MPI and FATmax, being this latter an index of aerobic exercise performance (Ishihara and Taniguchi, 2018). Future studies may support this influence of power on metabolic behaviour, with muscle power being responsible of the rightward and upward shift in the oxidation curves, therefore, higher MFO, CHOox_peak_ and a more delayed _rel_FATmax. Future studies should also analyse this behaviour in older female populations with even lower fitness level, despite the complexity of protocols that guarantee viable intensities in these unfit populations will be a big challenge. The role of muscle power goes beyond maximal metabolic intensities.

To conclude, several limitations should be recognized. The lack of baseline indirect calorimetry measurements, in addition to preventing us from knowing the oxidation of substrates in resting conditions, prevented us from calculating of net efficiency, an index of great relevance when talking about efficiency and mitochondrial content. According to Broskey et al. (2015), net efficiency, which has shown to be the more linked parameter with mitochondrial function, would have helped to better analyse the association between MFO and efficiency. A prior determination of VO_2_max would have also helped with questions arising FATmax and the efficiency outcomes affected by this parameter. On the other hand, the absence of mitochondrial measurements, as muscle fibers biopsies, prevented us from outlining the mechanism behind these findings (although these are invasive procedures). Future studies should include these more precise measurements to verify our results regarding MF in older women. Furthermore, due to the relevance of power and training status, we believe that it would be of interest to reproduce our study stratifying different cohorts according to these parameters.

## Conclusion

Despite being active, women over-60 confirmed impaired substrates switching and impoverished metabolic capacity in response to exercise, both, from FAT and CHO pathways. Nonetheless the confirmation of this deterioration process, it is not aging that determines the drop in MFO and CHOox_peak_, but muscle power. It suggests that those women who preserve power and reach higher performance in a cycling grade test, also preserve the ability to oxidize more fat at lower intensities, and a better switch to carbohydrates at the higher ones. Associated positively with power and inversely to gross efficiency, CHOox_peak_ has shown to be very informative in this indirect assessment of mitochondrial health, complementary to MFO.

Gross efficiency points to be the more appropriate variable in the assessment of mechanical efficiency in our study, showing independence from power, and an inverse association to carbohydrates oxidation which could be protective against the older women impoverished metabolic response. Similarly to young active cyclists, active old women increased efficiency independently from muscle power with the increase of intensity, although the former displayed this increased efficiency up to VT_1_, and the old women near VT_2_ (in absence of lower load intensities).

To conclude, our study suggests that the metabolic inflexibility with aging is highly dependent of fitness level in the form of muscle power, being this latter the most relevant variable for understanding metabolic flexibility in a population of active older women over-60.

## Data Availability

The raw data supporting the conclusions of this article will be made available by the authors, without undue reservation.

## Abbreviations

BLa, Blood lactate; CHOox, Carbohydrates oxidation rate; CHOoxpeak, Maximal Carbohydrates oxidation; DE, Delta efficiency; EC, Economy; FATmax, exercise intensity that elicits the MFO, expressed as a percentage of the VO_2_max; FATox, Fat oxidation rate; FFM, Fat free mass; GE, Gross efficiency; HR, Heart rate; IPAQ, International physical activity questionnaire; MF, Metabolic flexibility; MFO, Maximal fat oxidation; MPI, Metabolic power input; P60, Power at 60% of Powerpeak; P80, Power at 80% of Powerpeak; P100 or Powerpeak, Peak of muscle power at 100% of the test; PAR-Q, Physical activity readiness questionnaire; relFATmax, exercise intensity that elicits the MFO, expressed as a percentage of the VO_2_peak; RER, Respiratory exchange ratio; RPE, Rating of perceived effort; VAS, Visual analogue scale; VO_2_max, maximal oxygen uptake; VT2, Second ventilatory threshold.
